# Supplementary material for: Targeted Genomic Profiling and Chemotherapy Outcomes in Grade 3 Gastro-Entero-Pancreatic Neuroendocrine Tumors (G3 GEP-NET)
Source: Diagnostics (Basel). 2023 Apr 29;13(9):1595. doi: 10.3390/diagnostics13091595 (PMC10178589; doi:10.3390/diagnostics13091595)
Supplement: Supplementary file 1 [file diagnostics-13-01595-s001.zip › diagnostics-2353902-supplementary.pdf]

Supplementary Figure S1

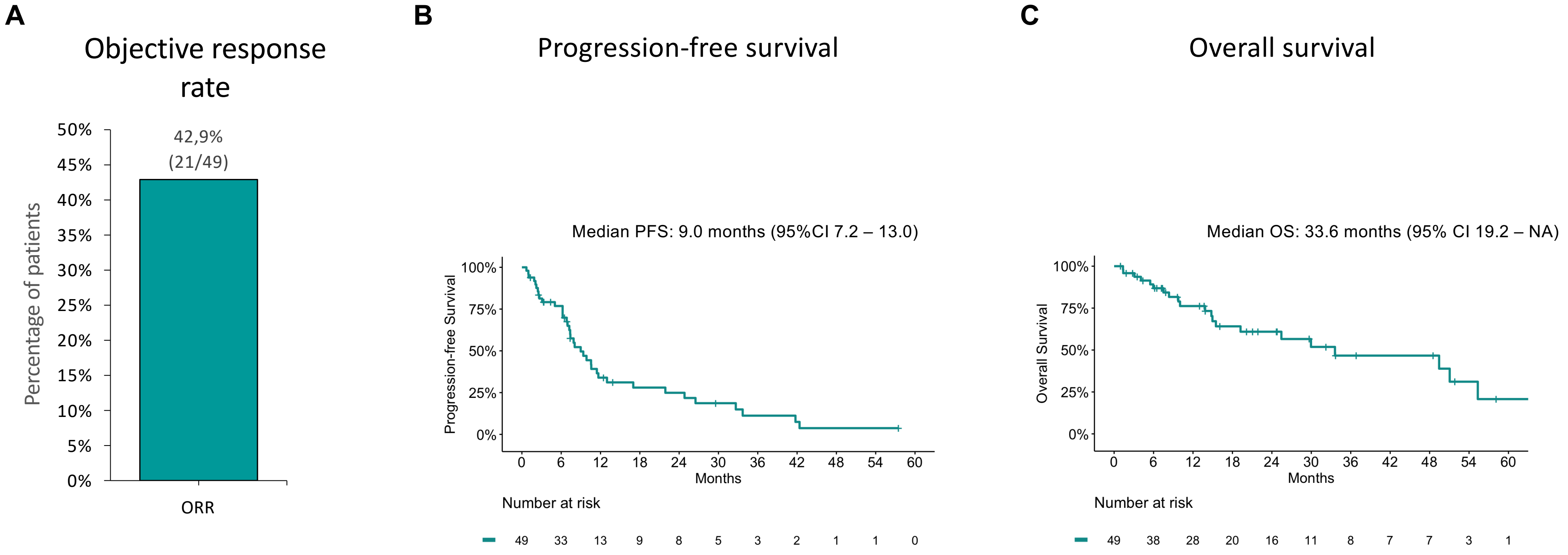

Supplementary Figure S2

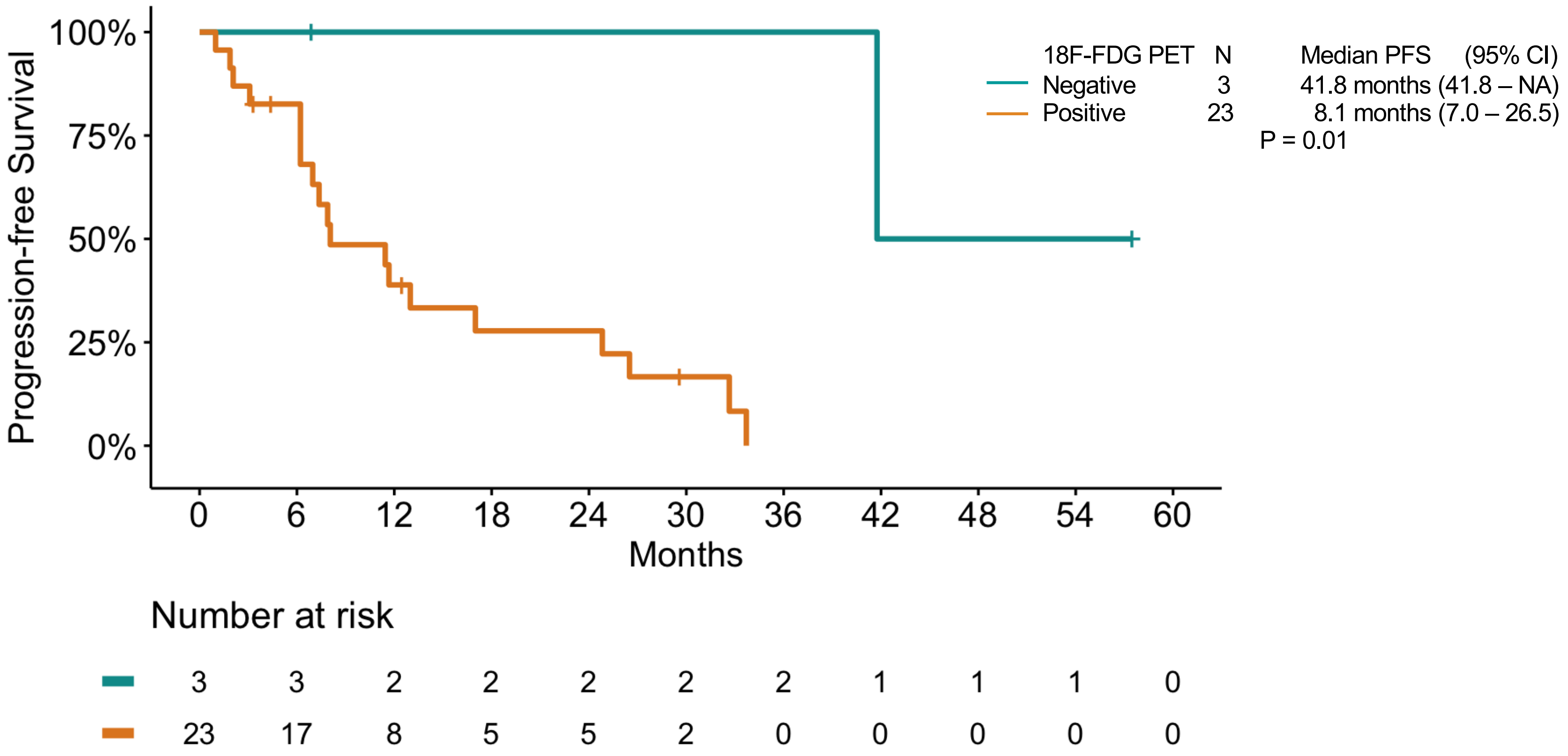

Supplementary Figure S3

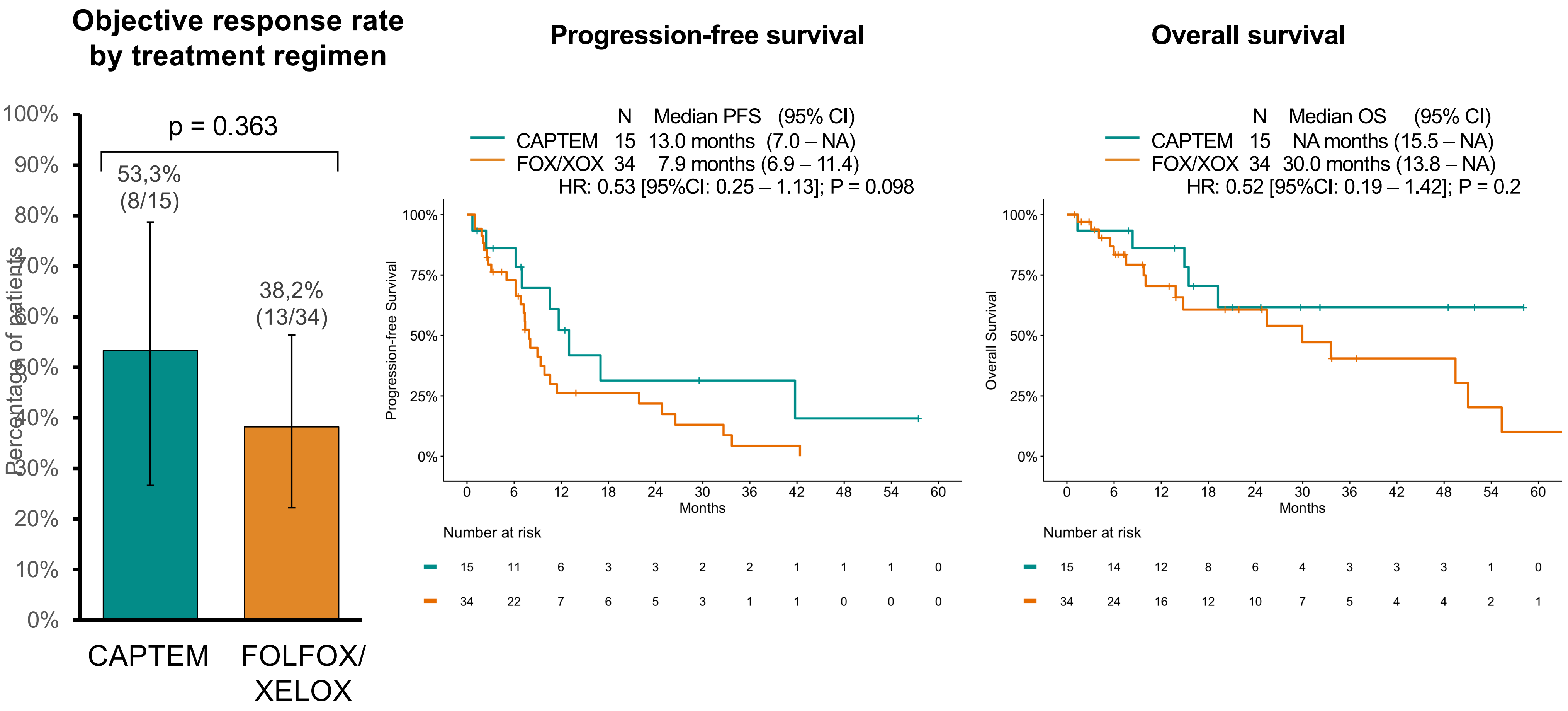

Supplementary Figure S4

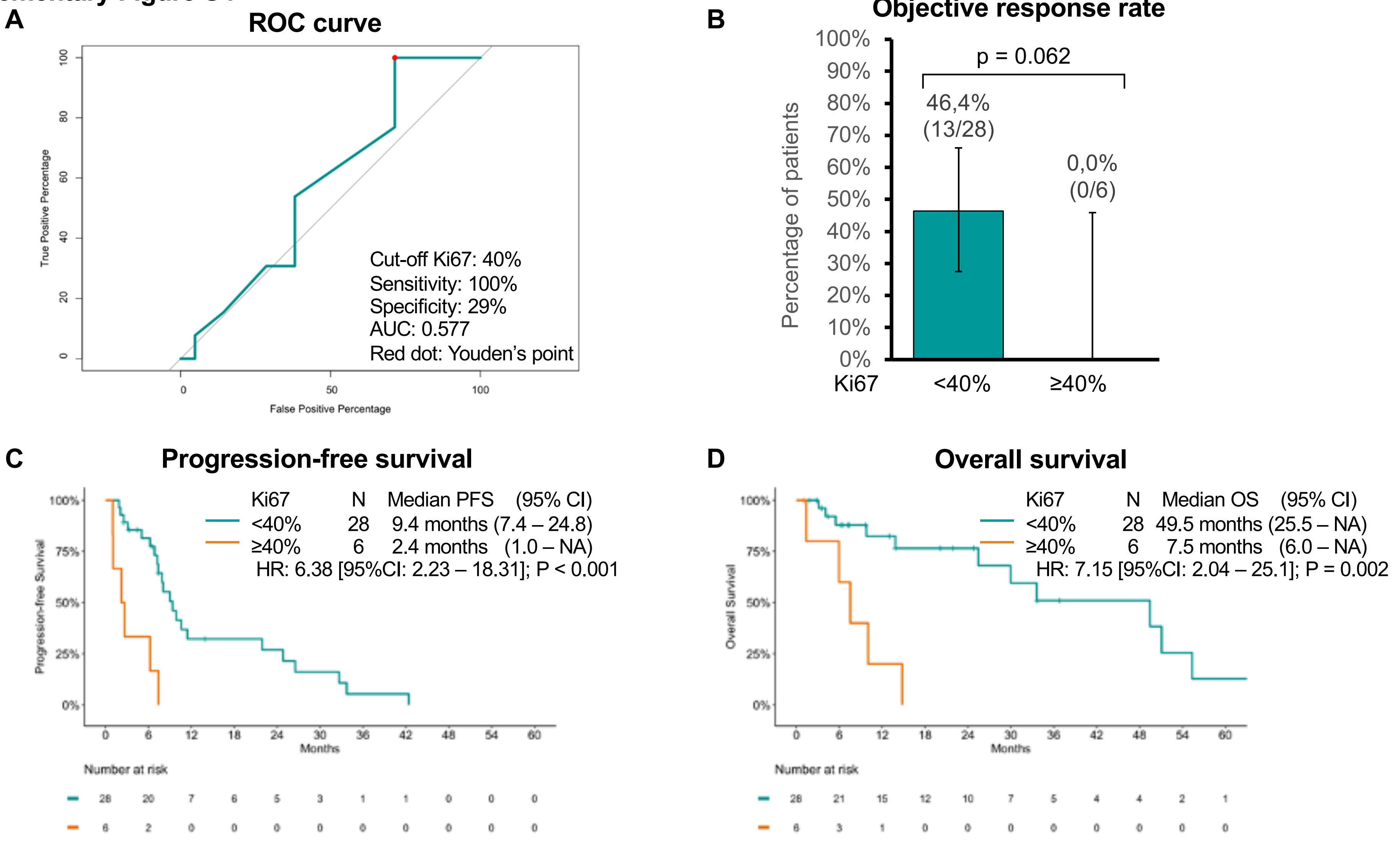

Supplementary Figure S5

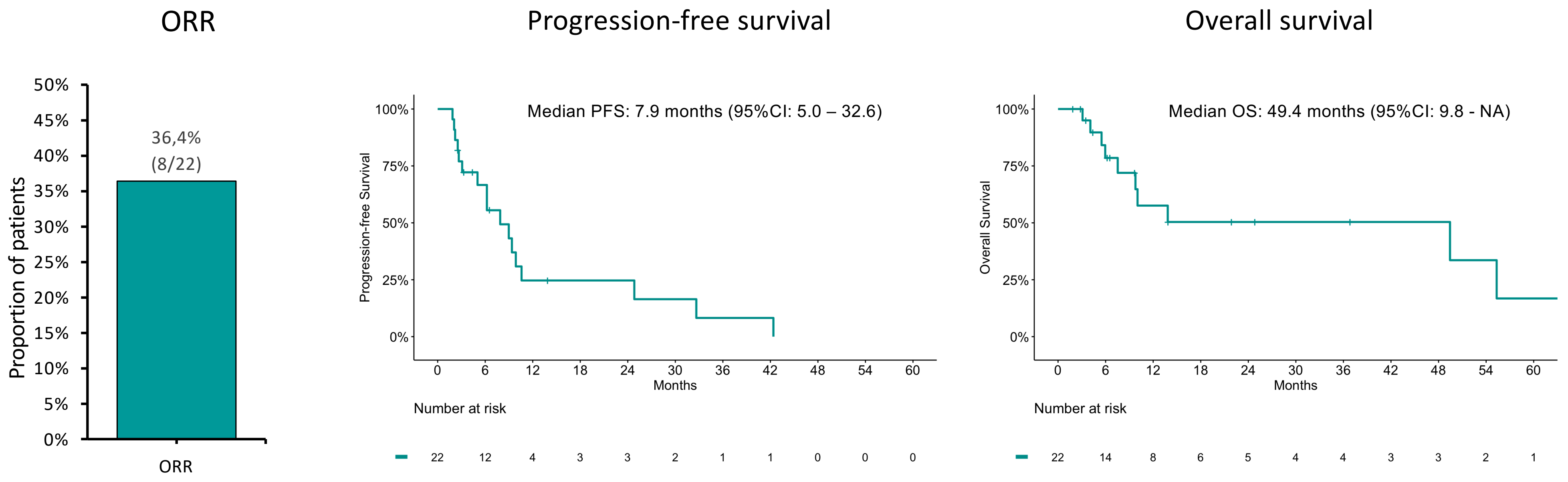

Supplementary Table S1. Cox proportional-hazards model for the risk of progression in the overall chemotherapy cohort. Only variables with a p-value <0.10 at the univariate model have been included in the multivariable model.

Variable	Univariate			Multivariate		
	HR	95%CI	P	HR	95%CI	P
Age*	1.01	0.98 – 1.03	0.673			
Primary site pancreas (vs GI)	0.70	0.36 – 1.37	0.298			
Stage IV (vs IIIB)	1.10	0.33 – 3.62	0.879			
18FDG-PET	NE	NE	NE	-	-	-
68Ga-DOTANOC PET	1.58	0.64 – 3.92	0.324			
SSA	0.91	0.47 – 1.76	0.780			
FOLFOX/XELOX (vs CAPTEM)	1.9	0.89 – 4.07	0.098	2.07	0.96 – 4.47	0.064
Reduced starting dose	1.69	0.85 – 3.34	0.132			
Ki67*	1.07	1.01 – 1.12	0.016	1.07	1.02 – 1.13	0.01

95%CI: 95% confidence interval; GI: gastrointestinal tract; PET: positron emission tomography; SSA: somatostatin analogues
*continuous variables
